# Olfactory cues elicit species-specific locomotive responses in poison frog tadpoles

**DOI:** 10.17912/micropub.biology.001532

**Published:** 2025-05-27

**Authors:** Mina E. Phipps, Penelope R. Baker, Luise Bachmann, Soyeon Park, Malia J. Perez, Shay Nair Sharma, Yvette Soto-Hernandez, Malaya Gaerlan, Marco Carrillo, Sofia Ceva, Sowmya Chundi, Binta Diallo, Juliana N. Fong, Kelly Huang, Jennifer Jackson, Jasmine Padilla, Leslie Quintana, Katelyn Santa Maria, Sadie M. Sarkisian, Paloma R. Sequeira, Eva U. Tatlock, Bryan H. Juarez, Najva Akbari, Max Madrzyk, Lauren A. O'Connell

**Affiliations:** 1 BIO161 Organismal Biology Lab, Stanford University, Stanford, California, United States; 2 Department of Biology, Stanford University, Stanford, California, United States

## Abstract

Amphibian species rear their larvae in distinct environments that may influence how they respond to different sensory stimuli. Here, we investigated the olfactory-mediated locomotive responses of two poison frog species ( *Allobates femoralis* and *Ranitomeya imitator* ) that vary in life history strategies. We found that *A. femoralis* tadpoles spent more time near an injury cue compared to control, while *R. imitator* tadpoles increased their movement in response to high concentrations of amino acids. These experiments were done in an undergraduate laboratory course, demonstrating how simple behavior assays conducted in a classroom setting can provide practical research experiences and new insights into animal behavior.

**Figure 1. Allobates femoralis tadpoles spend more time near injured conspecific cues and Ranitomeya imitator tadpoles increase their movement in response to amino acids.**
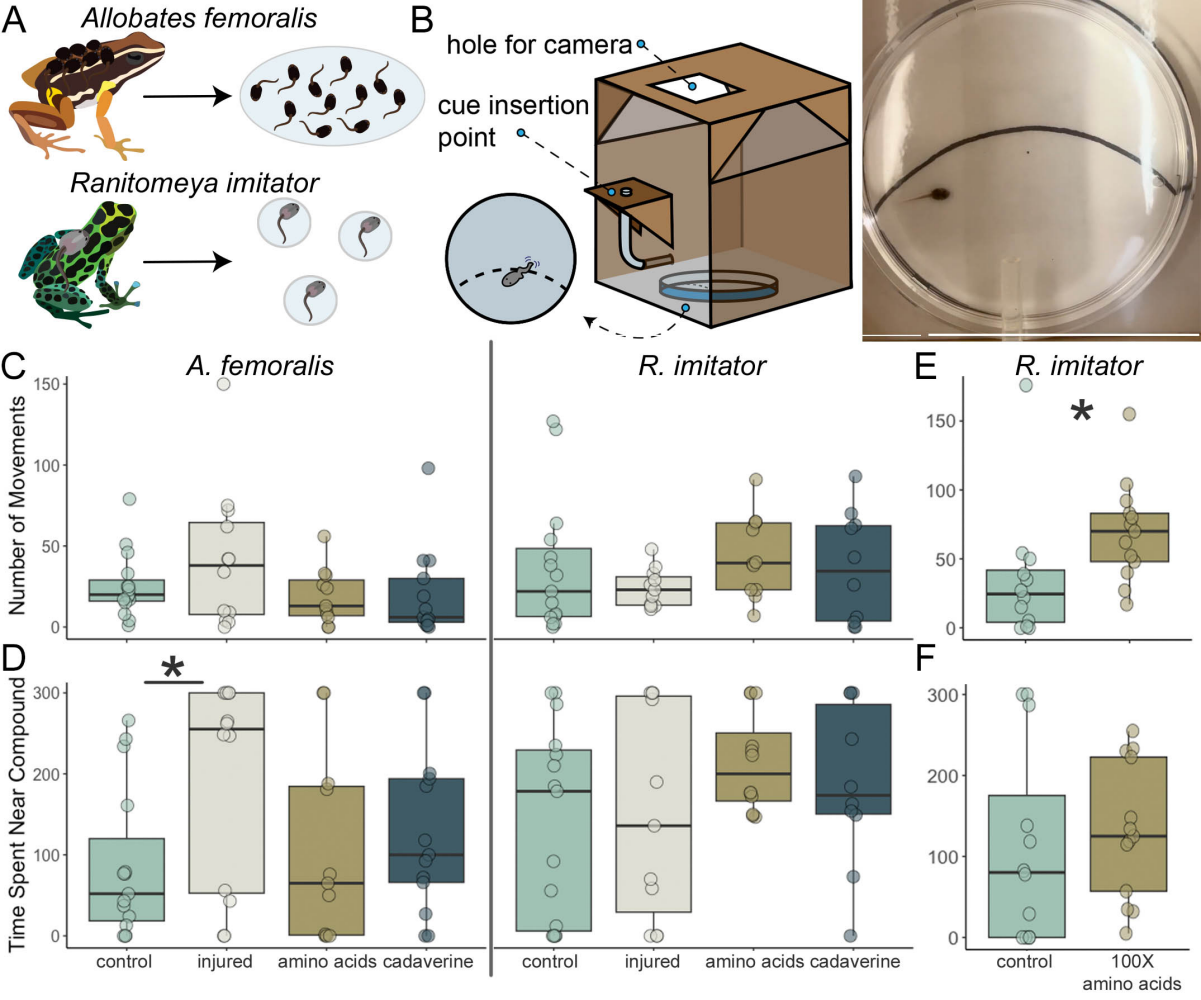
**(A)** *Allobates femoralis* tadpoles are transported in groups and live together in larger pools, while *R. imitator* are transported individually and live in isolation. **(B)** Diagram of apparatus used to administer compounds and record tadpoles during behavior trials. Right panel shows a video frame of the behavior assay. The black curved line divides the Petri dish arena in half. **(C)** Number of tadpole movements from *A. femoralis* (left) and *R. imitator* (right) in response to different compounds. **(D)** Time spent near the compound stimulus for *A. femoralis* (left) and *R. imitator* (right) tadpoles. *Allobates femoralis* spent significantly more time near the injury cue than control (t(46) = 2.620, p = 0.036). **(E)** *Ranitomeya imitator* tadpoles moved significantly more in response to 100x the initial amino acid concentration (GLMM, χ²(1) = 12.969, p <0.001). **(F)** Time spent for *R. imitator* tadpoles on the half of the arena near the 100x amino acid cue was administered.

## Description

Amphibians rear their larvae in diverse environments, which can shape how species perceive and respond to different sensory cues (Furness et al., 2022). Chemosensory systems in tadpoles are crucial for behavior, serving as an important pathway driving responses to environmental stimuli (Weiss et al., 2021). Understanding behavioral variation in how tadpoles respond to chemical information may provide insight into how differences in life history shape behavior and underlying neural systems. Here, we studied tadpoles of two poison frog species (Family Dendrobatidae) that have distinct rearing environments (Figure A). *Allobates femoralis* tadpoles develop in groups within larger pools and are not aggressive (Fouilloux et al., 2021). In contrast, *Ranitomeya imitator* tadpoles are individually raised in bromeliad pools, are fed unfertilized eggs by their mother, and display conspecific cannibalism (Brown et al., 2010, 2008). These species differences are linked to parent-offspring interactions during tadpole transport, and may have evolved due to natural selection related to carnivorous shifts in tadpoles that led parents to place tadpoles in isolated pools. Alternatively, these species differences could be the consequence of ecological selection where tadpoles were placed in isolated nurseries as parents shifted toward using new ecological niches in low-resource environments (Alonso-Alvarez and Velando, 2012). *Allobates femoralis* tadpoles experience predation from both insect and heterospecific predators (Szabo et al. 2021), whereas *R. imitator* are less likely to encounter these threats and are aggressive to other tadpoles deposited in their pools.

Given these species differences in life history, we hypothesized that tadpoles from each species would respond differently to various chemical cues associated with injuries, tissue decay, and/or food due to their different experiences as predator or prey. It is not understood how these species respond to various ecologically relevant odors and our study seeks to address this gap in knowledge by (1) using assays to compare behavioral responses to chemical stimuli in *A. femoralis* and *R. imitator* tadpoles and (2) carrying out these experiments in an undergraduate laboratory classroom to provide hands-on research experiences in animal behavior. We predicted that *A. femoralis* would respond to injury cues and tissue decay by exhibiting negative chemotaxis and moving less, whereas the predatory *R. imitator* would show positive chemotaxis and move more. We predict that both species would exhibit more movement in response to food cues.

To explore how chemical stimuli influence tadpole behavior, we assessed the locomotive responses of *A. femoralis* and *R. imitator* tadpoles to different chemical cues: a cue from an injured conspecific, an amino acid mixture, and cadaverine. Cadaverine, which signals decaying tissue, and cues from an injured conspecific typically elicit fleeing or freezing behavior in other aquatic organisms (Gonzalo et al., 2012; Kermen et al., 2020; Speedie and Gerlai, 2008). Amino acids signal potential food and elicit positive chemotaxis behavior in other aquatic organisms (Yang et al., 2024). We placed tadpoles in a Petri dish arena, added chemical cues, and measured the time spent within the cue zone (Figure C) and number of movements (Figure D). We chose these two measurements to understand if tadpoles are either attracted or repelled to the odors by recording what zone they spend time in and to measure their activity by recording bouts of movement. The omnibus test of an overall group/treatment/cue effect was insignificant for both species. The lack of significance for *A. femoralis* was likely a type II error resulting from the lack of statistical power associated with lower sample sizes. While the overall group effect was not significant when comparing what zone *A. femoralis* preferred (GLMM, χ²(3) = 7.451, p = 0.059), a pairwise test found that they spend more time near the injury cue than the control cue (Fig 1D, t(46) = 2.620, p = 0.036).

Our findings for the injury cue are consistent with previous research that also report varying responses to cues from injured conspecifics between species (Lipkowski et al., 2024). The latter responses may be associated with different ecological pressures faced by each species in their respective rearing environments (Ferrari et al., 2012, 2010; Phung et al., 2020; Płaskonka et al., 2024). The larger rearing pools for *A. femoralis* may expose them to higher competition and predation risk (Fouilloux et al., 2021), and the cue of an injured tadpole may indicate the presence of predators (Bairos-Novak et al., 2019). Thus, it may seem counterintuitive for *A. femoralis* tadpoles to move *toward* the stimulus. One interpretation is that this behavior is investigative, supporting previous research that tadpoles acquire and retain information about predators (Ferrari et al., 2012). Alternatively, injured or deceased tadpoles may serve as a food source, consistent with instances of opportunistic cannibalism in dendrobatid tadpoles (Caldwell and de Araújo, 1998; Dugas et al., 2016; Márquez, 2024; Summers, 1999), although this behavior would be more expected for the cannibalistic *R. imitator* tadpoles. However, because we raised *R. imitator* tadpoles in isolation, they may have not experienced the environmental cues that facilitate detection and response to conspecific injury (Regnet et al., 2023). Previous work has also found that tadpoles respond to a combination of visual and chemical predator cues (Szabo et al., 2021) and further work could test the behavioral response to injury cues in the presence of a conspecific tadpole to understand if bimodal cues cause a more robust behavioral response. Additionally, we did not include a heterospecific injury cue in our experiment, so we cannot disentangle whether the tadpoles are responding to injury cues generally or cues of a conspecific. Further experiments using heterospecific injury cues, varying stimuli concentrations, rearing conditions, and species would help determine the importance of positive and negative chemotaxis in relation to the behavioral ecology of cannibalism.

Cadaverine and amino acids did not elicit significant behavioral effects in either species in these initial experiments. Perhaps the tadpoles did not perceive the cues or recognize the cues as significant environmental indicators. We were particularly surprised by the lack of response to amino acids, as prior literature has demonstrated that *Xenopus laevis* tadpoles are attracted to amino acid odorants that are associated with food (Hassenklöver et al., 2012), leading us to investigate if the behavioral response is concentration-dependent. Given that *R. imitator* tadpoles did not spend more time near the amino acid cues relative to the control, we conducted a follow-up experiment where we increased the amino acid concentration to 100X the previous dose and again tested behavior for *R. imitator* tadpoles (Figure E-F). We observed a significant increase in movement (GLMM, χ²(1) = 12.969, p = 0.0003), suggesting the response of *R. imitator* tadpoles to amino acids is concentration-dependent. It is unclear whether these behavioral responses are based on olfactory or gustatory detection, as both systems can detect amino acids in other aquatic organisms (Gordon and Caprio, 1985; Hassenklöver et al., 2012; Vogler and Schild, 1999). Additional ablation experiments would be required to determine how tadpoles sense these chemicals in their environment. Further study into the relevant concentrations or biochemical and neuronal machinery used to detect these compounds in tadpoles is needed.

In summary, our study demonstrates species differences in chemosensory behaviors of *R. imitator* and *A. femoralis* tadpoles. These behavioral differences may reflect the ecological pressures experienced in communal pools versus isolated nurseries. Taken together, our results indicate that future studies should investigate species differences in responses to a range of compounds with varying concentration levels. This study also lays a groundwork for investigating the neural underpinnings of species differences in chemosensory processing. Finally, this work highlights the value of using simple behavior assays in undergraduate research courses, providing students with research experience while enhancing understanding of animal behavior and sensory ecology.

## Methods

*Experimental Animals and Housing*

*Allobates femoralis* and *Ranitomeya imitator* tadpoles were captive-bred in our animal colony and housed in individual containers during behavioral testing. Tadpoles were fed brine shrimp flakes on Mondays and Fridays and tadpole pellets on Wednesdays (Josh’s Frogs, Owosso, MI, USA) and underwent a full water change once per week. Additionally, each tadpole had access to sphagnum moss and tea leaves as extra sources of nutrients. All tadpoles used were early to middle stage (Gosner stages 25–30). Tadpoles were used in three behavior experiments in class with one week between experiments. One behavior experiment was conducted outside of class between weeks two and three. All procedures were approved by the Stanford University Animal Care and Use Committee (protocol no. 34429) **.**

*Behavior Assays*

We developed a custom behavior arena for testing tadpole responses to chemical stimuli that could be easily deployed in an undergraduate classroom (Figure B). A 10 cm Petri dish was filled with 40 mL of reverse osmosis conditioned DI water ("frog water", treated with Josh’s Frogs Dechlorinator Tap Water Conditioner) and placed on top of a laminated piece of paper containing a circle to line up with the Petri dish. We marked a curved line that transected the arena surrounding the cue deposition site following liquid-liquid radial diffusion principles (Watson, 1964). A custom laser-cut box (12.7 cm width x 14.6 cm length x 16.5 cm height) made from 31 mm medium-density fiberboard was placed over the top of the Petri dish and laminated paper. This box had a 5 cm square hole on the top so that a phone camera could be placed above the Petri dish and behavioral trials could be recorded. Additionally, one end of a 3 x 5 mm pure silicone tube was fixed to the exterior of the box, and the other was threaded through a hole in the wall of the box so that it was directly above the odor half of the petri dish.

Tadpoles were placed into the arena, allowed to acclimate for 2 min, and then baseline behavior was recorded for five minutes. Then, 1 mL of chemical odor was inserted into the silicone tube followed by 1 mL of water to ensure that all of the odor was deposited into the Petri dish. Behavior was recorded for an additional 5 min. We also conducted a colored dye trial to see how fast cues might disperse in our arenas. We found that the dye extended past the halfway zone in 2.4 seconds and across the whole arena in 22.8 seconds. However, because we do not know how fast our experimental odors disperse through the water, this may not be representative of the stimuli tested here. Following the initial trial, the water in the arena was replaced with 40 mL of new frog water, and tadpoles rested for 10 min. After the rest period, tadpoles were tested again using a different chemical odor. Individual tadpoles were given a maximum of two chemical odors per day and, at most, four distinct odors over the whole experiment. The time that each tadpole spent in the odor half of the arena and the number of movements the tadpole made within the 5-min trial were recorded. We defined a single movement as an event where a tadpole initiated motion and then came to a stop. Previous work hypothesized that by measuring movement, we can understand if tadpoles are exhibiting a search-like behavior or potentially hiding and not moving (Surber-Cunningham et al., 2024).

*Chemical compounds*

The chemical stimuli used in this experiment include cadaverine (0.1 mM in amphibian ringer solution, Sigma-Aldrich, D22606), a low and high concentration amino acid mix (1 mM or 100 mM of lysine, methionine, leucine, and arginine diluted in amphibian ringer solution, Sigma-Aldrich L5501, M5308, L8912, and A5006, respectively), amphibian ringer solution (control), and injured conspecific odors. We chose these amino acids because they are detected by the olfactory system in *Xenopus* tadpoles (Manzini et al., 2007; Terni et al., 2017) and because methionine and arginine are used as food cues in other aquatic organisms (Kermen et al., 2020). Cues representing an injured conspecific were prepared by anesthetizing tadpoles in 4℃ frog water until unresponsive to touch and then euthanized by cervical transection (Gonzalo et al., 2010). A chemical anesthetic was not used as these may interfere with behavioral trials. Whole tadpole bodies were homogenized in a 2 mL tube with 1.5 mm Zirconium beads along with 1 mL of amphibian ringer solution. Following tissue homogenization, tubes were centrifuged at 16000 rpm for 2 min, and supernatant was diluted in amphibian ringer solution such that the supernatant from one tadpole was contained in 4 mL of amphibian ringer solution. *Allobates femoralis* tadpoles were presented with injured *A. femoralis* cues and *R. imitator* tadpoles were presented with *R. imitator* cues. In total, 18 *A. femoralis* tadpoles (14 as experimental animals and 4 as injured conspecific stimulus) and 19 *R. imitator* tadpoles (15 as experimental animals and 4 as injured conspecific stimulus) were used.

*Data analysis*

Data analysis and visualization were performed in R version 4.4.0 (R Core Team, 2024). To ensure consistency with behavioral scoring, three students were compensated for scoring all behavioral videos outside of class, and the median values for each tadpole behavior assay were used for data analysis. Some trials were removed from the dataset due to errors in the assays, such as phones turning off and chemicals not delivered properly. We used a generalized linear mixed model (GLMM) in glmmTMB version 1.1.9 (Brooks et al., 2017) to separately regress time spent near the injured conspecific cue and the number of movements for each species onto the treatment group (the fixed effect). We included tadpole ID and week as random effects and removed them as necessary when their effects were at or near zero and caused convergence issues. Our GLMMs followed a normal residual distribution except for when modeling the number of movements that *A. femoralis* tadpoles made. We used the Akaike Information Criterion (AIC) to compare model fits among candidate residual distributions including the Poisson, Gaussian, and Negative Binomial distributions when modeling the number of movements for *A. femoralis* and found the Negative Binomial was the best fit. We confirmed appropriate model diagnostics using DHARMa version 0.4.6 (Hartig, 2024). We used the contrast function in the emmeans package version 1.10.2 (Lenth, 2024) to conduct a post-hoc pairwise test as necessary and p-values were adjusted for multiple comparisons using the false discovery rate correction. Data from our follow-up experiment was similarly cleaned and analyzed for each separate response variable. Boxplots for all experiments were generated using ggplot2 version 3.5.1 (Wickham, 2016), and the behavioral arena figure was created in Adobe Illustrator (version 2025).

*Classroom pedagogy*

The experiments in this study were performed over three laboratory sessions. The first behavioral trial was preceded by a demonstration by the instructors. Students worked in pairs, where one student recorded time spent on the odor side of the arena while the other student recorded the number of movements. Weekly homework included reading relevant literature, analysis and visualization of data collected by classmates, and writing an individual draft of a journal-style article that was combined into this article.

## Reagents

**Table:** 

**Strain Name**	**Genotype**	**Source**
Wildtype *Ranitomeya imitator* tadpoles	Wild type	O’Connell Laboratory at Stanford
Wildtype *Allobates femoralis* tadpoles	Wild type	O’Connell Laboratory at Stanford

**Table:** 

**Chemical Cue**	**Chemical composition**	**Source**
L-Arginine	H _2_ NC(=NH)NH(CH _2_ ) _3_ CH(NH _2_ )CO _2_ H	Sigma-Aldrich
L-Leucine	(CH _3_ ) _2_ CHCH _2_ CH(NH _2_ )CO _2_ H	Sigma-Aldrich
L-Lysine	H _2_ N(CH _2_ ) _4_ CH(NH _2_ )CO _2_ H	Sigma-Aldrich
L-Methionine	CH _3_ SCH _2_ CH _2_ CH(NH _2_ )CO _2_ H	Sigma-Aldrich
Cadaverine	NH _2_ (CH _2_ ) _5_ NH _2_	Sigma-Aldrich
Injured Conspecific Cue	Homogenized *A. femoralis* and *R. imitator* tadpoles	O’Connell Laboratory at Stanford
